# Breast cancer and dietary fat quality indices in Iranian women: A case–control study

**DOI:** 10.3389/fonc.2022.993397

**Published:** 2023-01-20

**Authors:** Fatemeh Shafie, Shirin Tajadod, Zahra Aslany, Pooneh Allahyari, Mahsa Vahdat, Soheila Shekari, Golsa Khalatbari Mohseni, Maryam Gholamalizadeh, Saeideh Mohammadi, Bojlul Bahar, Hanieh Shafaei, Saeid Doaei

**Affiliations:** ^1^ Nutrition Research Center, School of Nutrition and Food Sciences, Shiraz University of Medical Sciences, Shiraz, Iran; ^2^ Department of Nutrition, School Of Public Health, International Campus, Iran University of Medical Sciences, Tehran, Iran; ^3^ The Ohio State University Interdisciplinary ph.D. program in Nutrition (OSUN), Columbus, United States; ^4^ The Ohio State University Comprehensive Cancer Center, Columbus, United States; ^5^ Department of Exercise Physiology, Faculty of Physical Education and Sport Sciences, Islamic Azad University, Central Tehran Branch, Tehran, Iran; ^6^ Aboozar Children’s Medical Center, Ahvaz Jundishapur University of Medical Sciences, Ahvaz, Iran; ^7^ Department of Nutrition, Science and Research Branch, Islamic Azad University, Tehran, Iran; ^8^ Department of Nutrition, School of Allied Medical Sciences, Ahvaz Jundishapur University of Medical Sciences, Ahvaz, Iran; ^9^ Cancer Research Center, Shahid Beheshti University of Medical Sciences, Tehran, Iran; ^10^ Department of Nutrition, Zanjan University of Medical Sciences, Zanjan, Iran; ^11^ Nutrition Sciences and Applied Food Safety Studies, Research Centre for Global Development, School of Sport and Health Sciences, University of Central Lancashire, Preston, United Kingdom; ^12^ Nursing and Midwifery School, Guilan University of Medical Sciences, Rasht, Iran; ^13^ Department of Community Nutrition, Faculty of Nutrition and Food Technology, National Nutrition and Food Technology Research Institute, Shahid Beheshti University of Medical Sciences, Tehran, Iran

**Keywords:** breast cancer, dietary fats, fatty acids, cancer, fat

## Abstract

**Background:**

The association between breast cancer (BC) and different indices of dietary fats has not been well-studied. Thus, this study aimed to investigate the association between BC and dietary fat quality (DFQ) indices in Iranian women.

**Methods:**

This case–control study was conducted on 120 women with breast cancer and 240 healthy women in Tehran, Iran. Food Frequency Questionnaire and nutritionist IV software were used to assess the intake of dietary fats and to calculate the DFQ indices.

**Results:**

The patients with BC had a higher total fat (TF) (*P* < 0.01) and a lower ratio of polyunsaturated fatty acids (PUFAs) omega-3 to PUFAs omega-6 (ω-3/ω-6) compared with the controls (*P* < 0.001). TF had a significant association with BC risk (OR: 1.16; 95% CI: 1.01–1.33, *P* < 0.001). No significant association was found between BC and PUFA/saturated fatty acid ratio or the ω-3/ω-6 ratio.

**Conclusion:**

The patients with BC had a lower ω-3/ω-6 ratio and a higher total dietary fat intake than the healthy women. Total dietary fat intake was also directly associated with the risk of BC. Thus, low-fat diets may have beneficial effects for BC prevention. Further longitudinal studies are warranted.

## Introduction

Cancer, which reduces life expectancy, is a leading cause of death worldwide. The World Health Organization (WHO, 2019) declared cancer as the leading cause of death before age 70 in 112 of 183 countries partly due to the sharp decline in mortality rates from stroke and coronary heart disease compared with cancer (1). Breast cancer (BC) has now surpassed lung cancer as the leading cause of cancer in 2020, with an estimated 2.3 million new cases, representing 11.7% of all cancer cases. In addition, BC is the fifth leading cause of cancer mortality, accounting for 685,000 deaths globally (2). BC affects one in every four women and kills one in every six women worldwide. BC has been reported to be the most common cancer in Iran. According to the latest Iranian national database, the age-standardized rate for BC is 33.21 per 100,000, with an overall 5-year survival rate of 72% in women and 60% in men. The mortality rate is 14.2 per 100,000, and the 5- and 10-year survival rate is 81% and 77%, respectively (3, 4).

Many factors could be associated with BC (5, 6). Among the various risk factors identified, diet plays an important role in the incidence of BC (7–9). Despite being extensively studied, only a few dietary components associated with the incidences of BC have been identified (6)—for instance, dietary patterns characterized by a low intake of fruits, vegetables, and whole grains and high in red meats, saturated fat, and sodium are reported to be associated with a higher risk of developing BC (10). The underlying mechanisms of dietary components and their mechanistic role in the molecular as well as cellular pathways associated with the BC pathway are yet to be elucidated (6).

The results on the association between dietary fat intake and BC risk were controversial. High intakes of dietary fat have been linked to an increased risk of BC in adult women (7, 8). However, some prospective cohort studies (4), meta-analysis studies (11), and observational studies have reported a weak (12–14) or a non-significant relationship between BC and dietary fats (7, 15–17). Furthermore, the association between the type of fat consumed and the development of BC is not yet clear. Farvid et al. (18), as well as Tayyem et al. (8), reported that a higher intake of saturated fatty acids (SFA) and monounsaturated fatty acids (MUFAs) was associated with an increased risk of BC. In contrast, two observational studies (19, 20) reported a protective effect of MUFAs on the development of BC. In addition, several studies reported that polyunsaturated fatty acid (PUFA) intake increased the risk of BC (7, 8, 12, 21). However, this result was not confirmed by a case–control study (22) and a meta-analysis (11). Since different dietary fatty acids are metabolized through common pathways and the amount of intake of one group of fatty acids may be effective on the metabolism of other fatty acids (11), it is possible that considering them separately does not present the correct result in terms of their connection with BC. Hence, this study aimed to investigate the association between BC and different types of dietary fat quality indices in Iranian women.

## Methods

### Participants

This case–control study was performed in September 2020 on 120 women with BC and 240 healthy age-matched women referred to the cancer clinic of Shohadaye Tajrish Hospital, Tehran, Iran. A 1:2 case‐to‐control ratio was used in this matched case–control study due to concern for sufficient numbers in the stratified analysis and increase in power given the expected prevalence of exposure among the controls. The inclusion criteria for the case group were as follows: women with BC, age between 35 and 65 years, no more than 1 month after the diagnosis of BC, no diseases affecting food intake, and no dietary fat or fatty acid supplement intake. The inclusion criteria for the control group were as follows: age between 35 and 65 years, no disease affecting food intake, no use of dietary fat or fatty acid supplements, and do not have any type of cancer. The exclusion criteria were the inability to collect the required information and any conditions that may affect the diet during the last year. Written informed consent forms were obtained from all participants before the study.

Data on age, number of pregnancies, breastfeeding duration, number of abortion cases, family history of BC, and physical activity were collected. Anthropometric indices including weight, height, body mass index (BMI), and waist circumstance were collected.

### Dietary fat quality indices

A validated semi-quantitative Food Frequency Questionnaire (FFQ) was used to assess the dietary intake over the past year through face-to-face interviews with a trained dietitian (12). The amounts of intake of different types of dietary fatty acids were assessed using Nutritionist IV software (version 3.5.2, Axxya. Systems, Redmond, WA, USA), modified for Iranian foods.

Energy percentage from total fat (TF) was calculated by dividing the average obtained energy from fat (kcals) by the average daily energy intake. Furthermore, other dietary fat quality (DFQ) indices were calculated as follows: PUFA-to-SFA ratio (PSR) was calculated by the amount of dietary intake of PUFAs divided by the amount of dietary intake of SFAs. The ω-3/ω-6 ratio was also calculated by the amount of dietary intake of omega-3 fatty acids divided by the amount of dietary intake of ω-6 fatty acids (13).

### Statistical analyses

Data analyses were conducted using version 26 of Statistical Package Software for Social Sciences (SPSS Inc., Chicago, IL, USA). The Kolmogorov–Smirnov’s test and a histogram chart were used to test the normality of the data. Baseline characteristics and dietary intakes were expressed as mean ± SD or median (interquartile range, IQR) for quantitative variables with normal and skewed distribution, respectively. Independent sample *t*-test and Pearson chi-square test were applied to compare the quantitative and qualitative variables between the groups, respectively. A comparison of the medians between the two groups was done using the Mann–Whitney *U*-test. Binary logistic regression was then utilized to estimate the odds ratios (ORs) and 95% confidence intervals (CIs) adjusted for multiple covariates in different models. The significance level was determined as *P <*0.05.

## Results

In this study, 360 participants (120 cases and 240 controls) were included. More than 93% of the participants (*n* = 335) were menopausal women. The general characteristics and the dietary intakes of the case and the control groups are presented in **Table 1**. The median (IQR) age of the participants in the cases and the controls was 58.50 (51.50, 66.75) and 48.00 (42.25, 56.00) years, respectively (*P* < 0.001). The two groups had significant differences in BMI (<0.05), breastfeeding duration (*P* < 0.001), and family history of BC (*P* < 0.001). Compared with the control group, BC patients consumed significantly more calories (*P* < 0.001), carbohydrates (*P* < 0.001), and total fat (*P* < 0.001).

**Table 1:** Characteristics of the study population.

	Cases (*n* = 120)	Controls (*n* = 240)	*P*_value_
Age (years)^a^	58.50 (51.50–66.75)	48.00 (42.25–56.00)	<0.001^x^
BMI (kg/m^2^)^b^	27.63 ± 4.48	28.88 ± 4.35	0.05^†^
Breastfeeding duration (months)^a^	24.00 (10.50–48.00)	54.00 (39.50–74.00)	<0.001^x^
First menstruation (years)^a^	13.00 (12.50–14.00)	13.00 (12.00–14.00)	0.77^x^
Postmenopausal age (years)^a^	49.00 (43.50–52.00)	48.00 (44.00–51.00)	0.65^x^
Family history of BC^c^	22 (7.4%)	21 (7.0%)	<0.001
Number of pregnancies^a^	3.00 (2.00–4.00)	3.00 (2.00–5.00)	0.07^x^
Smoking^c^	3 (1.0%)	2 (0.7%)	0.05^¶^
Physical activity (h/day)^a^	1.00 (0.75, 1.25)	1.00 (0.25, 2.00)	0.92^x^
Energy (kcal/day)^a^	2,551.98 (2,454.20– 2,643.84)	1,862.63 (1,408.26–2,471.02)	<0.001^x^
Carbohydrates (g/day)^a^	362.27 (345.94–383.53)	245.18 (167.05–341.32)	<0.001^x^
Total fat (g/day)^a^	91.07 (83.19–100.04)	70.08 (53.13–90.36)	<0.001^x^
Proteins (g/day)^b^	81.32 ± 16.65	75.00 ± 32.33	0.15^†^

BMI, body mass index; BC, breast cancer.

a N (range).

b Mean (SD).

c N (%).

**Figure 1:**
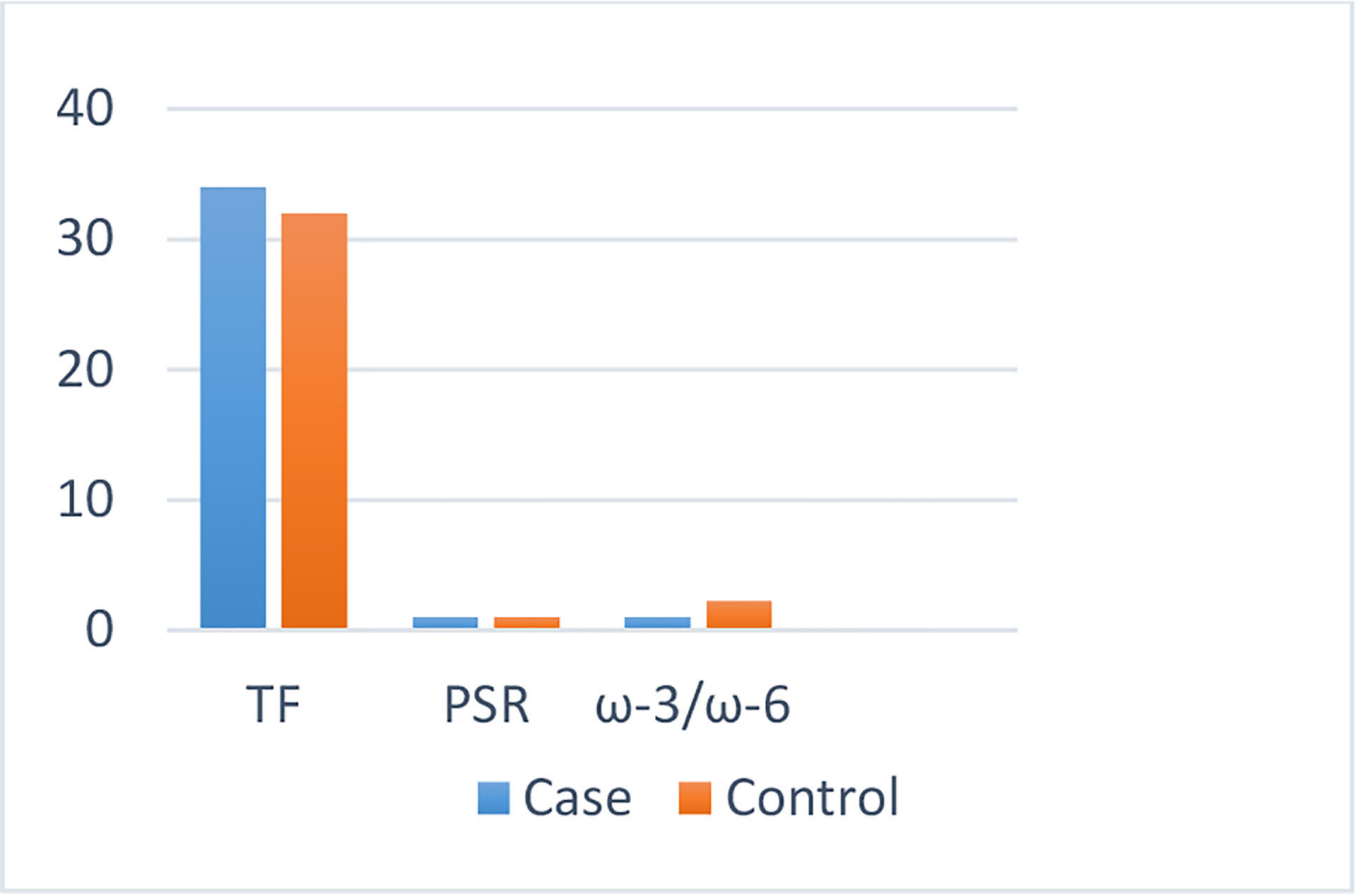
Dietary fat quality indices of the study population.

The dietary fat quality indices among the case and the control groups (**Table 2**) indicated that individuals with BC obtained higher amounts of energy from consuming fat (*P* < 0.01) and that their diet had a significantly lower level of ω-3/ω-6 compared with those of the controls (*P* < 0.001) (**Figure 1**).

**Table 2:** Dietary fat quality indices of the study population.

	Cases (*n* =120)	Controls (*n* = 240)	*P*_value_
TF^a^	34.68 (29.20, 42.24)	32.33 (30.01, 34.64)	0.002^b^
PSR^a^	0.65 (0.47, 1.06)	0.65 (0.48, 0.94)	0.68^b^
ω-3/ω-6^a^	0.21 (0.10, 0.76)	1.25 (0.76, 5.13)	<0.001^b^

TF, energy percentage from total fat; PSR, polyunsaturated fatty acids/saturated fatty acids ratio; PUFA, polyunsaturated fatty acids.

a Values are median (interquartile range).

b P-values obtained using Mann–Whitney U-test.

**Figure 2:**
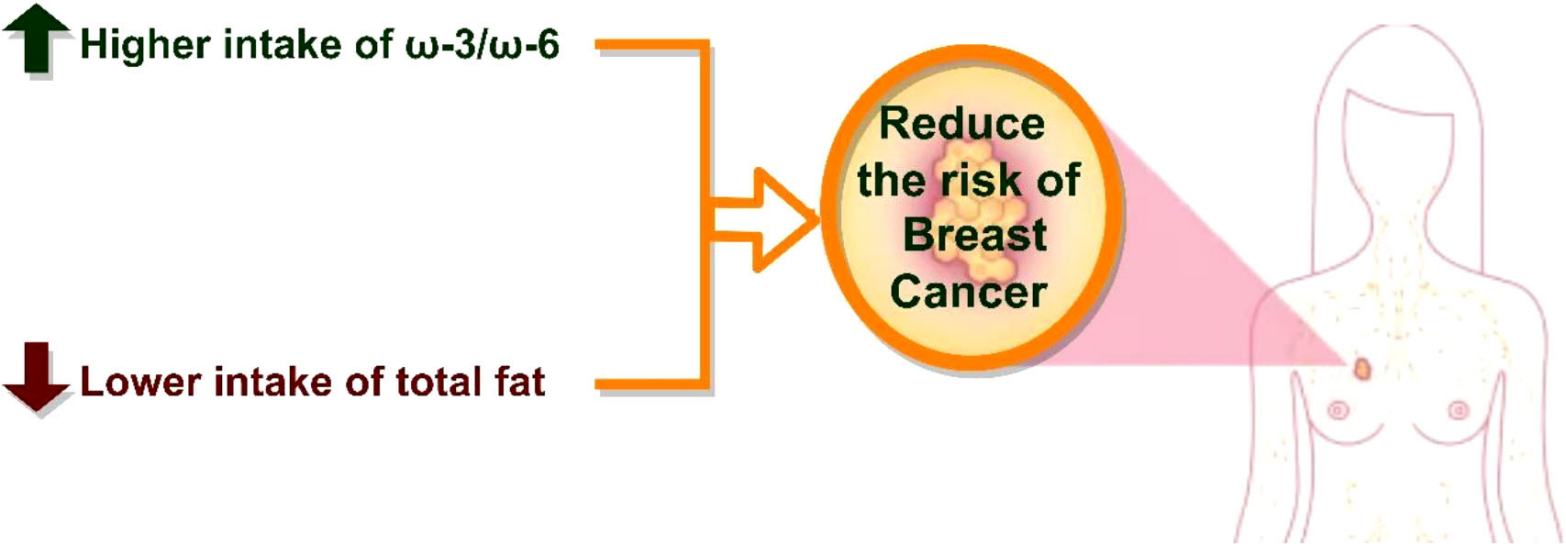
The association between breast cancer and dietary fat.

The crude and adjusted ORs (95% CIs) for the underlying associations between TF, PSR, plus ω-3/ω-6 ratio, and BC risk are reported in **Table 3**. Individuals with a higher TF had greater odds for BC (OR: 1.16; 95% CI: 1.01–1.33), though no association between the PSR and ω-3/ω-6 ratio with BC risk was found (**Figure 2**).

**Table 3:** Logistic regression of the association between dietary fat quality indices and breast cancer.

Variables	OR	95% CI	*P*
TF			
Model I^a^	1.09	(1.04, 1.15)	<0.001
Model II^b^	1.16	(1.01, 1.33)	0.03
PSR			
Model I^a^	0.88	(0.55, 1.40)	0.60
Model II^b^	1.15	(0.69, 1.90)	0.58
ω-3/ω-6			
Model I^a^	1.01	(0.97, 1.04)	0.55
Model II^b^	1.009	(0.92, 1.10)	0.84

TF, energy percentage from total fat; PSR, polyunsaturated fatty acids/saturated fatty acids ratio; PUFA, polyunsaturated fatty acids; OR, odds ratio; CI, confidence interval.

a Crude association.

b Adjusted for age, body mass index, energy intake, number of pregnancies, breastfeeding duration, number of abortion cases, family history of breast cancer, and physical activity.

## Discussion

Despite the heavy interest in investigating the association between fat intake and BC, their relationship is still controversial. The aim of the current study was to explore the relationship between dietary fat quality and BC in Iranian women. In this study with 120 cases and 240 controls, it was observed that individuals with BC had a higher intake of total fat and a lower ω-3/ω-6 ratio compared with the controls, and the risk of developing BC was positively associated with the total fat intake. There was no association between BC risk and the polyunsaturated-fat-to-saturated-fat ratio or ω-3/ω-6 index.

Several case–control studies have reported a positive association between total fat intake and BC risk (14–17), while prospective cohort studies have not confirmed these observations (7, 18). In other words, previous studies have shown no association between total fat and BC, or this positive association has been trivial (23). These conflicting results may be due to the different assessment methods in different studies—for example, it has been suggested that the use of food records may provide a stronger correlation between dietary fat and BC risk compared with the FFQs (19, 20). Furthermore, several studies have reported that non-dietary risk factors such as menopausal status, a history of benign breast disease, and menopausal hormone therapy may affect the association of total fat and BC (7, 14, 21, 22, 24).

In our study, the polyunsaturated-fat-to-saturated-fat ratio was similar in both groups, while the ω-3-to-ω-6 ratio was higher in the cases than in the controls. Similar to our findings, some previous studies have found no association between BC and fat subtypes (25–27). However, contrary to the findings of our study, one study reported a significant positive association between fat subtypes and the risk of BC in postmenopausal women (17), and some other studies reported an association between BC and animal fat (a rich source of SFA) in premenopausal women (27–29). One of the reasons for the discrepancy observed in the results of different studies may be the difference in menopausal status. Moreover, the effect of dietary fat on the risk of BC may be influenced by the level of sex hormones.

Furthermore, the results of the present study indicated that the difference of the ω-3/ω-6 ratio between cases and controls was significant; still no association was found between this ratio and the risk of BC. According to previous studies, PUFAs may have different effects on the risk of BC based on the double bond position (30, 31). Some studies have established that PUFAs, particularly linoleic acid and arachidonic acid, promote while marine-derived ω-3 fatty acids inhibit mammary tumorigenesis (17, 31, 32). In addition, some case–control studies have found a positive association between ω-6 fatty acid intake and the risk of BC (33, 34). On the other hand, another study reported that serum ω-6 fatty acids were inversely related to BC risk (35). Based on the report of cohort studies, ω-3 fatty acids have protective effects on BC (36, 37). Furthermore, other studies showed an inverse relationship between ω-3 PUFAs and the risk of BC (38–40). However, in some other studies, ω-3 fatty acids did not affect BC (41–44). Because of these conflicting results, some researchers suggested that ω-3-to-ω-6 ratios should be used instead of a specific fatty acid (45). Two studies reported that the ω-3-to-ω-6 ratio is inversely related to BC (46–50). Some other studies of ω-3/ω-6 PUFAs and BC led to contradictory results (51, 52). The various results observed in different studies can be due to differences in the study population, dietary sources of specific fats, amounts of total fat and specific fatty acids, menopausal status, and methods used to measure fat intake. Thus, it is suggested that large-scale studies with different dietary patterns as well as well-designed trials be conducted to indicate the association between total fat and DFQ and the risk of BC.

This study suggested that energy percentage from total fat may be a more critical factor in determining the risk of BC. A large randomized, controlled trial reported that a low-fat diet might reduce the risk of developing BC in post-menopausal women (53). A meta-analysis study reported that the risk of BC is higher in post-menopausal women on a high-fat diet. At the same time, dietary fat may have protective effects in pre-menopausal women. Thus, capturing the menopausal status of people can also be a determining factor (54).

Several mechanisms have been proposed for the possible association between different types of dietary fats and BC. Previous studies have identified that SFAs may play a more significant role in determining the risk of BC than other types of fat (7, 25). SFAs may increase insulin resistance (55), where an association between plasma insulin concentration and the risk of BC has been reported (56). Dietary PUFAs usually contain a high proportion of linoleic acid, a precursor to prostaglandins (57). Arachidonic acid and prostaglandin E2 may play a role in inducing BC by affecting estrogen synthesis (48). It has been suggested that marine ω-3 may affect the BC risk, possibly through effects on BMI or related factors (insulin and adiponectin) (40). The mechanisms proposed for the protective effect of ω-3 PUFAs include suppressing the biosynthesis of arachidonic acid-derived eicosanoids, altering the estrogen metabolism, reducing the production of free radicals, and modifying insulin sensitivity (58). However, it should be noted that the association of ω-3 fatty acids with BC risk may be influenced by the availability of dietary antioxidants such as vitamins E and C (58). Moreover, ω-6 fatty acids are easily oxidized due to their high double bonds and thus contribute to cell damage. They may also increase the risk of BC by competing with ω-3 fatty acids in producing eicosanoids (33).

As in all case–control studies, the major limitation of the current study was recall bias. Women who are aware of a beneficial diet can often give overestimated amounts of products that have health-promoting effects. In addition, the evaluation of PUFAs with FFQ may differ on the actual content of the product, which may depend on different periods of the harvest of food products, storage time, or method of cooking (59). Furthermore, considering that the majority of the participants were menopausal, a comparison between menopausal and non-menopausal women was not possible. Future studies should attempt to determine the content of individual fatty acids in the patients’ foods and in their blood samples (60). The duration of breastfeeding in controls was also significantly higher than in the cases and in various studies; breastfeeding has been suggested as a protective factor against BC (61). There was a significant difference between the case and control groups regarding the family history of BC and genetic factors. Given the fact that the effects of these factors in the regression models were adjusted, the association between total fat and BC observed in this study is hence robust enough.

## Conclusion

This study suggested an association between the amount of dietary fat and the risk of BC. Patients with BC had a lower ω-3/ω-6 ratio in their diet and a higher intake of total dietary fat than others. As a result, a lower intake of dietary fats may be beneficial in BC prevention. Further studies are warranted to confirm these results and to identify the underlying mechanisms of the association between dietary fat and BC.

## Data availability statement

The original contributions presented in the study are included in the article/supplementary material. Further inquiries can be directed to the corresponding author.

## Ethics statement

All patients signed an informed consent form at baseline. This study was approved by the ethical committee of Shahid Beheshti University of Medical Sciences, Tehran, Iran (code: IR.SBMU.CRC.REC.1398.006). The patients/participants provided their written informed consent to participate in this study.

## Author contributions

FS, MG, SD, ZA, ST, MV and SS designed the study, involved in the data collection, analysis, and drafting of the manuscript. SD, MA, SM, BB, and MG were involved in the design of the study. All authors contributed to the article and approved the submitted version.
